# Multi-Target Regulation by Small RNAs Synchronizes Gene Expression Thresholds and May Enhance Ultrasensitive Behavior

**DOI:** 10.1371/journal.pone.0042296

**Published:** 2012-08-21

**Authors:** Jörn Matthias Schmiedel, Ilka Maria Axmann, Stefan Legewie

**Affiliations:** 1 Institute for Theoretical Biology, Charité-Universitätsmedizin, Berlin, Berlin, Germany; 2 Institute of Molecular Biology, Mainz, Rheinland-Pfalz, Germany; Université Paris Descartes; INSERM, U1002., France

## Abstract

Cells respond to external cues by precisely coordinating multiple molecular events. Co-regulation may be established by the so-called single-input module (SIM), where a common regulator controls multiple targets. Using mathematical modeling, we compared the ability of SIM architectures to precisely coordinate protein levels despite environmental fluctuations and uncertainties in parameter values. We find that post-transcriptional co-regulation as exemplified by bacterial small RNAs (sRNAs) is particularly robust: sRNA-mediated regulation establishes highly synchronous gene expression thresholds for all mRNA targets without a need for fine-tuning of kinetic parameters. Our analyses reveal that the non-catalytic nature of sRNA action is essential for robust gene expression synchronization, and that sRNA sequestration effects underlie coupling of multiple mRNA pools. This principle also operates in the temporal regime, implying that sRNAs could robustly coordinate the kinetics of mRNA induction as well. Moreover, we observe that multi-target regulation by a small RNA can strongly enhance ultrasensitivity in mRNA expression when compared to the single-target case. Our findings may explain why bacterial small RNAs frequently coordinate all-or-none responses to cellular stress.

## Introduction

Since their discovery more than thirty years ago, it has become clear that small RNAs (sRNAs) play a crucial role in regulating gene expression. sRNAs downregulate gene expression post-transcriptionally by pairing with target mRNAs through base complementarity. Complex formation with small RNAs competitively inhibits mRNA translation and/or induces mRNA degradation (reviewed in [1], [2]). Importantly, the interaction between the sRNA and its target is non-catalytic in nature, since sRNA molecules are typically degraded along with their target, instead of being re-used to regulate other targets [3]. Such regulation is distinct from other post-transcriptional regulators such as RNA-binding proteins (RBPs) and microRNAs (miRNAs) which, in most cases, pass through multiple rounds of mRNA complex formation [4]. On the other hand, RBPs and miRNAs can competitively inhibit translation, and thus resemble sRNA action [5], [6]. Notably, sRNA-mediated regulation conceptually differs from transcriptional repression, besides simply regulating a later step in protein biosynthesis: transcriptional repressors are typically present in vast excess over individual binding sites in the genome; thus, unlike sRNAs, the repressor pools are not depleted by binding to specific targets. Compared to other modes of regulation, sRNAs may thus confer unique dynamical features to gene expression.

The quantitative aspects of sRNA regulation were analyzed by various mathematical modeling studies, most of which assumed a purely stoichiometric mode of sRNA action [7], [8], [9], [10], [11], [12], [13], [14]. Model-based analyses revealed that sRNAs binding their targets with sufficiently high affinity can establish a threshold-linear gene expression response at steady state [9]: the stoichiometric nature of sRNA action ensures that mRNA translation is almost completely suppressed as long as the sRNA concentration exceeds that of the mRNA (sub-threshold regime). In contrast, gene expression increases linearly with increasing mRNA transcription as soon as the sRNA is less abundant than the mRNA species (linear regime). Recent work revealed that miRNAs can generate similar threshold-linear behavior at the single-cell level [15]. Regulation by sRNAs has a clear signature not only for steady state expression but also during dynamic responses. For example, the system may initially need to eliminate excess unbound sRNA when reaching the linear regime; thus the kinetic profile of gene expression is characterized by a sharp delay [7], [16].

Mathematical modeling studies revealed that steady state and temporal thresholds require that sRNAs bind strongly to their targets. Accordingly, some sRNA-mRNA complexes were shown to be stable and can be assumed to form irreversibly, as expected for sufficiently long RNA duplexes [17], [18], [19]. However, sRNA species vary as to the extent they complement their targets, and even in case of extensive complementarity base pairing may only occur over a limited region, the so called “kissing complex” [20], [21], [22]. In order to maintain their regulatory effects, many sRNAs require the presence of a specific RNA chaperone protein, Hfq, which is thought to melt inhibitory RNA structures and may have a bridging function in mRNA binding [22]. In fact, the presence of Hfq increases local concentrations of sRNAs and mRNAs that drastically enhances complex formation. For example, a 50-fold increase in mRNA-association rate in the presence of Hfq has been measured between *rpoS* mRNA and DsrA sRNA [23] or between *ompA* and MicA [24]. Thus, in living cells high affinity complex formation may be ensured by additional factors beyond simple base pairing. Notably, Hfq has also been implicated in protecting the sRNAs from degradation until paired and in recruitment of the degradosome to sRNA-mRNA complexes [22], suggesting intensive regulatory interactions between sRNAs and RBPs.

Small RNAs regulate a broad range of targets, many of which function during bacterial stress responses, e.g., by regulating outer membrane proteins, iron metabolism, quorum sensing or carbohydrate metabolism [22]. By being expressed only under specific conditions, sRNAs contribute to adaptation to environmental changes. On the other hand, constitutively expressed sRNAs appear to limit the expression of proteins that become toxic when expressed in large amounts [21], [22], [25]. Many sRNAs have the potential for interacting with multiple mRNAs, which allows them to coordinate whole physiological responses [26]. One of the first sRNAs observed to interact with more than a single mRNA target was DsrA in *E. coli* effecting translation of global transcription factors [27]. Another well-established sRNA example for controlling multiple targets is RyhB, which downregulates at least 18 operons encoding iron-using proteins [28]. Very recently, 16 regulated mRNA targets were identified for Spot42, a small RNA that modulates catabolite repression in *E. coli* [29]. For GcvB and RybB in *Salmonella* it has been demonstrated that a conserved domain of the sRNA is responsible for recognizing multiple mRNA targets encoding ABC transport systems or outer membrane proteins, respectively [30], [31]. A recent systematic target profiling and validation approach revealed RybB and MicA to act each as global mRNA repressors during envelope stress [32]. In summary, many sRNAs (e.g. RyhB, Spot42, RybB, MicA, CyaR, DsrA, GcvB, OmrAB, RNAIII) turned out to act as multiple-target regulators [26], [29], [32]. It is very likely that multi-target regulation by a small RNA is a recurrent design principle adding a new layer of complexity for bacterial gene expression. Multi-target regulation by a single sRNA was analyzed in previous mathematical modeling studies (reviewed in [33]): These papers showed that a single sRNA is able to efficiently downregulate many targets at once ([13], [34]), and that mRNAs crosstalk between each other by sequestering the common sRNA regulator [8]. Mitarai et al. proposed the concept of hierarchical prioritization, where RNAs respond sequentially to decreasing levels of sRNA expression, depending on their affinity for the common sRNA regulator [11]. In this paper, we extend these previous theoretical studies on sRNA-mediated multi-target regulation, and re-evaluate the parameter requirements of hierarchical prioritization by analytical and numerical studies.

**Figure 1:**
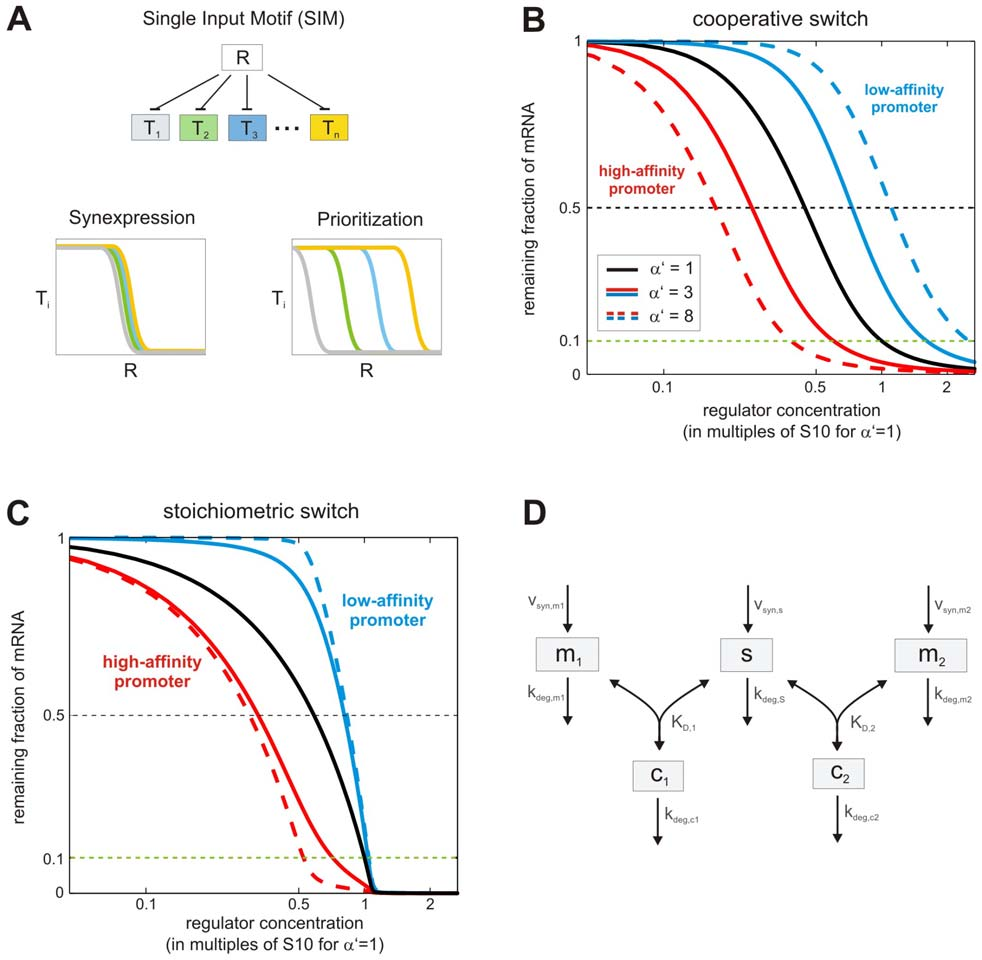
SIM architectures differ in their ability to robustly coordinate gene expression responses. (A) The single-input module (SIM), where a regulator (R) controls multiple targets (T_i_) may establish coordinated expression of all targets (synexpression) or sequential regulation if target affinities differ (prioritization). (B) Threshold of a cooperative repression model is sensitive to changes in regulator affinity. The steady state dose-response of the cooperative repression model (Eq. 1) was calculated while varying the ratio of promoter-regulator affinities α′ = K_D,2_/K_D,1_ (other parameters: V_max,i_ = 1; k_deg,i_ = 1; n = 3). The x-axis is normalized by the regulator concentration where the case α′ = 1 reaches 10% of maximal mRNA expression. (C) An extended cooperative repression model with regulator depletion is less sensitive to parameter alterations. The steady state dose-response of a mass-action model explicitly describing three sequential binding steps of the regulator to the two target promoters was calculated numerically (see Protocol S1 for details). This system is a generalization of the minimal cooperative repression model (Eq. 1). High affinity regulator binding to mRNAs was assumed to ensure that regulator depletion effects are significant. The x-axis is normalized by the regulator concentration where the case α′ = 1 reaches 10% of maximal mRNA expression to allow direct comparison with panel B. (D) Mathematical model for sRNA-mediated co-regulation. The mRNA species (m_1_ and m_2_) are controlled by reversible binding to the shared sRNA (s), giving rise to inhibitory complexes, c_1_ and c_2_. The monomeric species m_1_, m_2_ and s are constantly synthesized, and all molecular species are subject to degradation. Throughout this work we assume that the inhibitory complexes c_1_ and c_2_ are formed with high affinity.

Multi-target regulation systems (Fig. 1A, top) are recurrent motifs in biochemical networks, and are commonly referred to as “Single-input modules” (SIMs). Depending on the kinetic parameters, SIMs can show two types of behavior (Fig. 1A): (i) if all targets show similar affinity for the regulator, they may respond synchronously once a critical regulator concentration is reached. Such behavior has been termed “synexpression”, and is thought to require fine-tuning of target affinities [35]. (ii) In the other extreme, low-affinity targets may be relieved from inhibition at lower regulator concentrations than high-affinity targets. Experimental studies confirmed that such hierarchical prioritization occurs in transcriptional responses to morphogen gradients [36]. In this work, we compare different SIM architectures and parameter regimes, and show that some intrinsically favor synexpression while others tend to promote hierarchical prioritization. Since coordinated regulation of functionally related proteins is thought to optimize cellular responses [35], [37], [38], [39], [40], we focused on a SIM architecture that favors synexpression even without fine-tuning of target affinities: sRNA-mediated multi-target regulation. Using analytical approximations, we comprehensively characterize the parameter space, and show that robust synexpression at steady state is a general feature of the system, unless target affinities are extremely different. Numerical studies show that shared sRNAs are able to synchronize the temporal dynamics of mRNA expression as well; moreover, sRNAs can establish strong feedback regulation in larger networks. Taken together, we extend previous studies on mRNA crosstalk by sRNA sequestration [8], and show that sequestration effects may be beneficial in robustly synchronizing mRNA expression levels under various regulation regimes. Our findings suggest that post-transcriptional control may be more efficient in coordinating gene expression when compared to transcriptional modes of regulation.

## Results

### SIM architectures differ in their ability to robustly coordinate gene expression responses

Single-input modules (SIMs), where a single regulator controls multiple targets (Fig. 1A, top) are recurrent motifs in biochemical networks. SIMs may result in synexpression or prioritization of the regulator targets (Fig. 1A and Introduction). To better understand the requirements for synexpression and hierarchical prioritization, we analyzed the steady state behavior of simple SIM architectures by calculating the response to increasing regulator concentrations. We focused on regulation mechanisms controlling gene expression at transcriptional and post-transcriptional levels. Consider the following minimal model of cooperative transcriptional co-regulation, where a transcriptional repressor R_T_ inhibits transcription of the mRNA species m_1_ and m_2_ (1)
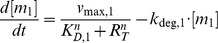


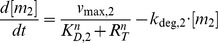
The kinetic parameters V_max_, K_D_, n and k_deg_ correspond to the maximal transcription rate, the dissociation constant of promoter binding, the Hill coefficient and the mRNA degradation rate, respectively. The steady state solution [m_i_] = V_max,i_/k_deg,i_/(K_D,i_^n^+R_T_^n^) implies that the repressor concentration for half-maximal inhibition equals K_D,i_. Thus, the threshold repressor concentrations eliciting half-maximal mRNA downregulation depend in a linear manner on the repressor affinity for the promoter. Moreover, the distance between the mRNA dose-response curves scales with α′ = K_D,2_/K_D,1_ (cf. Fig. 1B). Therefore, the transcriptional switch easily accommodates expression prioritization and sequential regulation, as even moderate affinity differences: (i) allow the two mRNAs to respond at significantly different regulator concentrations. (ii) establish an exclusive expression regime of the low-affinity mRNA at intermediate regulator concentrations (depending on the steepness of the dose-response curves). On the other hand, precise synexpression of the two mRNAs in the transcriptional switch can only be established for very similar affinities K_D,i_, and thus requires extensive parameter fine-tuning. In the following, we will discuss SIM architectures whose mRNA expression thresholds diverge in a less-than-linear manner with affinity differences. We will argue that this property promotes robust synexpression, independent of precise kinetic parameter values.

In Eq. 1, repressor pools are not depleted by binding to promoter sites; this assumption is likely to be fulfilled in transcriptional networks, where abundant transcriptional regulators (cf., [41]) target only two specific promoter copies per cell, and are buffered by non-specific binding to the genome. To understand the potential role of regulator depletion, we analyzed an extended cooperative inhibition model, where the repressor concentration is no longer assumed constant (see Protocol S1). The corresponding dose-response curves in Fig. 1C reveal that the distance between repression thresholds scales less than linearly with the affinity ratio α: for example, the threshold repressor concentrations eliciting half-maximal mRNA downregulation differ by ∼3-fold if the affinities differ eightfold (α = 8). Parameter insensitivity is even more pronounced in the lower part of the dose-response. In such a system, synexpression would require less extensive parameter fine-tuning, making it a more plausible mechanism for precise co-regulation. We conclude that some SIM architectures intrinsically favor synexpression while others tend to promote hierarchical prioritization. In the Supporting Information, we show that the partial threshold insensitivity observed in Fig. 1C is directly linked to regulator depletion and sequestration effects (see Protocol S1). Regulator depletion effects play a major role in post-transcriptional gene expression regulation [8], [33]; we therefore decided to comprehensively analyze the parameter (in)sensitivity in a minimal SIM, where a small RNA inhibits two mRNAs (Fig. 1D). We focused on a system where the sRNA is co-degraded with its targets, because such a mechanism favors synchronization and coupling effects when compared to a catalytic mode of action (cf. Discussion). It will be shown below that the thresholds of the sRNA system are even more robust than the ones in Fig. 1C.

### Modelling the regulation of multiple mRNAs by a shared sRNA

Post-transcriptional co-regulation of multiple mRNAs by a shared small RNA was analyzed using the model depicted in Fig. 1D. The translation of the mRNA species (m_1_ and m_2_) is competitively inhibited by the sRNA (s). Additionally, the effective turnover rate of the mRNA pools is controlled by the sRNA, since the degradation rates of the inhibitory complexes, c_1_ and c_2_, may differ from those of the free mRNA species. By employing the law of mass-action, the dynamics of post-transcriptional regulation can be described by the following set of differential equations (2)
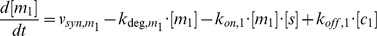


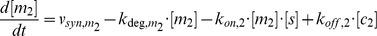


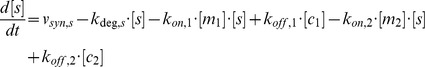


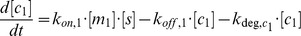


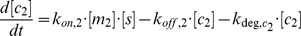
This system comprises 12 kinetic parameters, i.e., three synthesis rates (v_syn_), five degradation rate constants (k_deg_), and four rate constants describing complex association and dissociation (k_on_, k_off_).

Simplifying assumptions were made to derive analytical expressions for the dose-response behavior and parameter sensitivity of the system: It was assumed that the system has reached steady state, thus (initially) neglecting the temporal aspects of the response. As the steady state of the full system (Eq. 2) cannot be solved analytically, the affinity of the mRNA-regulator complexes, c_1_ and c_2_, was additionally assumed to be very high (K_d,i_ = k_off,i_/k_on,i_→0). As outlined in the introduction, this seems to be a reasonable assumption for many sRNA circuits. In case of strong binding, all mRNA will be bound into the inhibitory complexes (c_1_ and c_2_) as long as the sRNA is present in excess, i.e., (3)
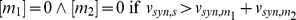
In the following, we will focus on the opposite regime (v_syn,m1_+v_syn,m2_>v_syn,s_) to understand principles governing the accumulation of free, translationally active mRNA.

### Analytical approximation for strong post-transcriptional co-regulation

At steady state, transcription balances RNA degradation, giving rise to the following set of algebraic equations (4)
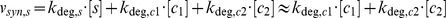


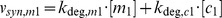


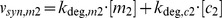
Degradation of free sRNA is neglected, since the whole sRNA pool will be bound completely into the complexes c_1_ and c_2_ as long as v_syn,m1_+v_syn,m2_>v_syn,s_. Taking into account the binding equilibria of the complexes (*K_d,i_^eff^* = (k_off,i_+k_deg,ci_)/k_on,i_ = [s]⋅[m_i_]/[c_i_]), one can solve for the steady state concentration of the free mRNA species (5)
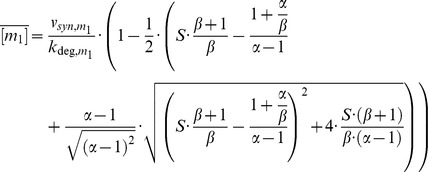


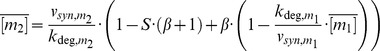
Importantly, the steady state of the 12 parameter system in Eq. 2 is fully determined by five lumped parameters (α, β, S, v_syn,m1_/k_deg,m1_, v_syn,m2_/k_deg,m2_). The mRNA synthesis ratio β relates the production terms of the two mRNAs (6a).
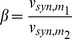
while the mRNA inhibition strength ratio α quantifies the sRNA-mediated inhibition of m_2_ relative to m_1_ (6b)
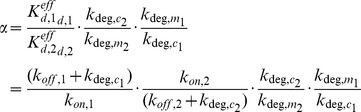
Large values of α imply that: (i) the sRNA affinity for m_2_ is larger than that of m_1_ (K_d,2_^eff^<K_d,1_^eff^) and/or (ii) sRNA-mediated destabilization of m_2_ is large relative to m_1_ (k_deg,c2_/k_deg,m2_>k_deg,c1_/k_deg,m1_). Thus, α>1 indicates that sRNA-mediated inhibition of m_2_ is stronger than that of m_1_. For equal inhibition of both mRNAs (α = 1) the steady state solution simplifies to (7)
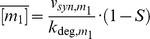


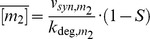
In Eqs. 5 and 7, the mRNA species simultaneously become positive if the stimulus S fulfills (8)
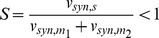
Thus, accumulation of free mRNAs can only occur if the sum of the mRNA transcription rates exceeds the sRNA transcription rate (v_syn,m1_+v_syn,m2_>v_syn,s_; cf. Eq. 3). Using numerical simulations of the full system (Eq. 2), we confirmed that Eqs. 5 and 7 approximate the steady state of the system very well as long as complex formation is sufficiently strong.

Equation 7 describes a threshold-linear response with a switch from no expression to linear expression at S = 1. Thus, for equal inhibition of both mRNAs (α = 1), each mRNA behaves similar to the case where a single mRNA is strongly inhibited in a threshold-linear manner by a small RNA [7], [9] or a miRNA [15].

### sRNA-mediated co-regulation can synchronize gene expression thresholds despite different affinities for individual mRNAs

**Figure 2:**
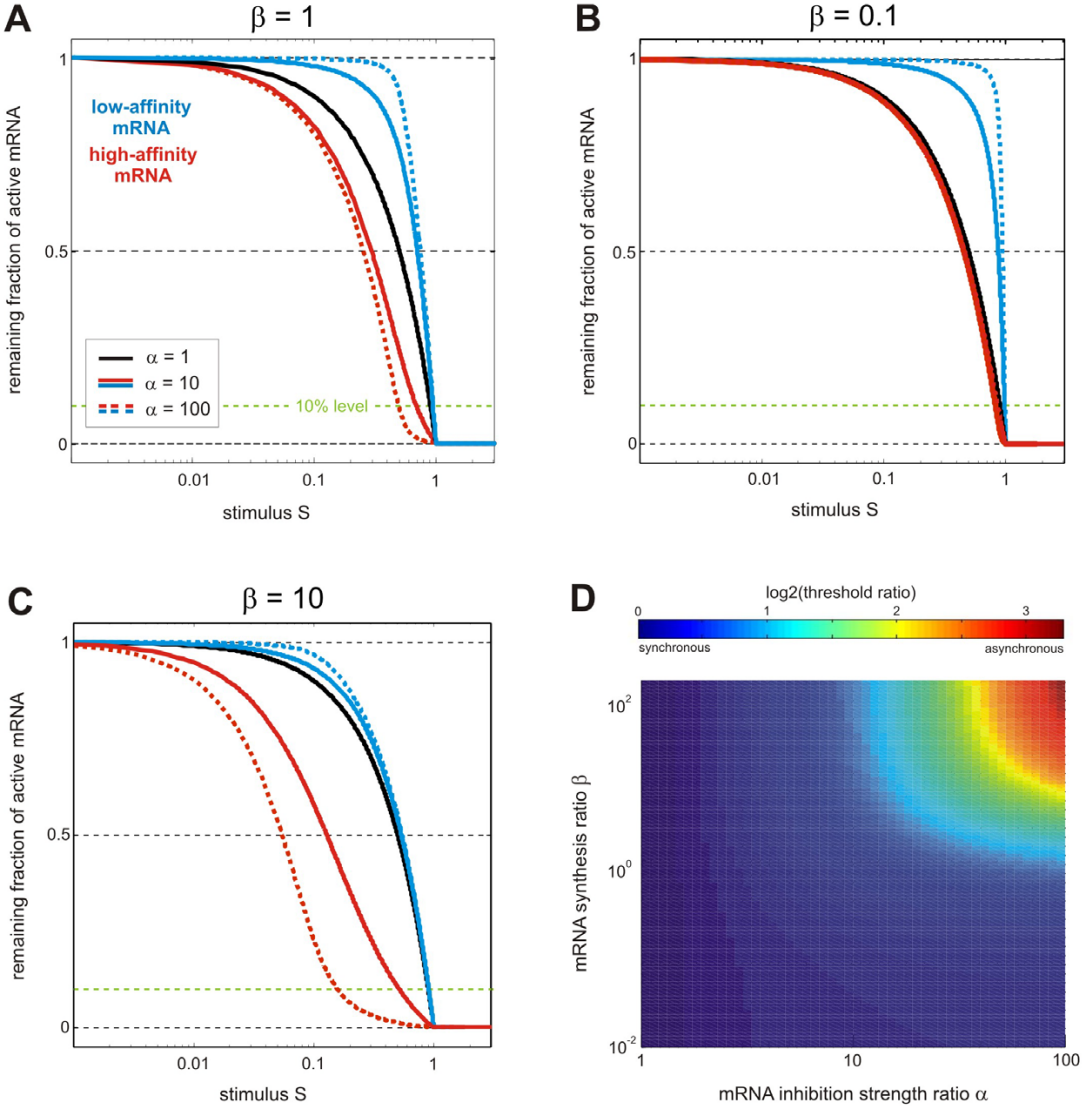
sRNA-mediated co-regulation can synchronize gene expression thresholds despite different affinities for individual mRNAs. (A) Steady state dose-response curves of mRNA expression for equal synthesis rates of both mRNAs (β = 1). The remaining fraction of active mRNA species (Eq. 9) is shown as a function of the normalized sRNA synthesis rate (stimulus S; Eq. 8). The dose-response curves of the two mRNAs completely overlap if both mRNAs are inhibited with the same efficiency (α = 1; Eq. 6; black line), while they diverge for increasingly different inhibition strengths (α = 10; α = 100). According to Eq. 5, the system behavior is solely determined by the lumped parameters α and β. (B) and (C) Steady state dose-response curves of mRNA expression (same as in panel A) for unequal synthesis rates of both mRNAs (β = 0.1 or β = 10; cf. Eq. 4). (D) Gene expression threshold synchronization occurs over a broad range of kinetic parameters. The log2 threshold ratio (Eq. 10) was calculated for varying mRNA inhibition strength (α) and synthesis ratios (β) using Eq. 5. Thresholds were calculated using the 10% stimulus levels (S10, cf. horizontal green line in panels A–C), and divided to calculate the threshold ratio (Eq. 10). Blue areas in the heatmap reflect synchronous switching of both mRNAs.

To analyze the parameter sensitivity of mRNA co-regulation, it is instructive to analyze the remaining fraction of active mRNA (9)
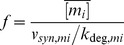
The free mRNA concentration in the absence of sRNA equals [m_i_] = v_syn,mi_/k_deg,mi_. Thus, the remaining fraction of active mRNA quantifies the percent inhibition of each mRNA by the sRNA. Plotting *f* as a function of the stimulus S (Fig. 2) yields doubly normalized dose-response curves fully characterizing mRNA regulation in terms of the constants α and β (Eq. 5). In biological terms, the stimulus S may correspond to alterations in the sRNA transcription rate (v_syn,s_).

For equal inhibition strength (α = 1), the mRNAs show perfect co-regulation, since the dose-response curves completely overlap regardless of the synthesis ratio β (Fig. 2 A, B, C; Eq. 7). Unequal inhibition strength (α≠1) results in preferential suppression of the high-affinity mRNA. Nevertheless, the gene expression thresholds of the system respond in a sub-sensitive manner to changes in α, thus supporting that the system favors synexpression: The stimuli of 10% or 50% mRNA expression (dashed horizontal lines in Fig. 2 A, B, C) can be used to quantify threshold positions in a robust manner. Even if strong affinity differences are allowed (α≤100) these threshold measures differ less than two-fold between the two mRNAs in most cases, and the ratio never exceeds a factor of 5 in Figs. 2 A, B, C. Thus, the thresholds of the sRNA circuit are even less parameter-sensitive than the thresholds of the cooperative switch with regulator depletion (Fig. 1C). Therefore, repression of the two mRNA typically occurs at very similar sRNA concentrations. Moreover, an exclusive expression regime for the low-affinity mRNA (‘sequential regulation’) can only be established for very large affinity differences (α≥100).

In the context of stress responses, it is most likely the all-or-none transition from no expression to a significant level that matters. In the following, we will therefore use the stimuli of 10% mRNA expression to systematically analyze alignment in this lower part of the dose-response. Synchronous switching is comprehensively quantified in Fig. 2D using the logarithmic difference between the 10% stimulus levels (S10) of both mRNAs (10).
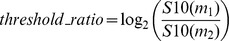
Alterations in the parameters α and β generally induce sub-sensitive changes in the threshold ratio, confirming that synchronous switching is a robust property of the system. Threshold de-synchronization is restricted to the upper-right region of the heatmap where the inhibition strength α and the synthesis ratio β are large (Fig. 2C and D). In this non-robust regime a highly abundant weak affinity mRNA and a low abundance high affinity mRNA coexist. Once the sRNA concentration is too low to inhibit both mRNAs (i.e., if S<1), the high affinity mRNA pulls the regulator away from the low affinity mRNA. Thus, the low affinity mRNA starts to be freed from the sRNA, while the high affinity mRNA is still subject to inhibition, giving rise to two distinct thresholds (dashed lines in Fig. 2C). It should, however, be noted that the thresholds still desynchronize in a sublinear manner even in this most sensitive regime.

Besides transcriptional induction of the sRNA, the circuitry in Fig. 1 may also be controlled by alterations in the expression of one or both mRNAs, raising the question of whether synchronous switching is maintained under these conditions. The dose-response curves in Fig. 2 are based on the assumption that α and β are constant, and therefore continue to hold for co-linear regulation of both mRNA transcription rates (v_syn,s_ = const.; v_syn,m2_ = β • v_syn,m1_ with β = const.). Thus, a constitutively expressed sRNA synchronizes gene expression thresholds arising from transcriptional co-regulation of the two mRNAs. Moreover, parameter-independent coordinated switching is also maintained for selective transcriptional regulation of one mRNA (e.g., v_syn,s_ = constant; v_syn,m1_ = constant; v_syn,m2_≠const; Protocol S2). This is due to the fact that high-affinity sRNAs serve as push-pull devices that couple the expression of an unregulated target mRNA with that of a regulated target mRNA. For equal affinity of both mRNAs (α = 1; Eq. 7), the strength of this push-pull effect can be quantified by calculating the gain of the free m_1_ concentration with respect to the synthesis rate (11)
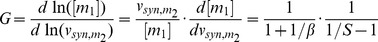
Thus, for the case where m_2_ transcription is regulated, the expression of m_1_ can respond infinitely strong if the system is close to the threshold (S≈1). The effect is enhanced if m_2_ is more abundant than m_1_ (β>1). The former condition explains our observation that gene expression thresholds are coupled, while the upper parts of the dose-response curves are less well coordinated.

Taken together, we comprehensively characterized the parameter space to show a shared sRNA establishes synchronous gene expression thresholds irrespective of the mode of transcriptional regulation in the circuit. Numerical simulations confirm the synchronization effect, but also reveal that the effect is less pronounced if the low-affinity mRNA binds too weakly to the sRNA (non-stoichiometric mode of inhibition): in this case, large sRNA concentrations are required to fully repress the low-affinity mRNA, and, as a result, the threshold ratio would be higher than predicted by the analytical approximation.

### sRNA-mediated co-regulation may convert threshold-linear mRNA dose-response curves into a very steep all-or-none switch

Our simulations in Fig. 2B revealed that the low-affinity mRNA can exhibit an extremely steep dose-response curve for β = 0.1 and α≥10. To understand this effect intuitively, we will analyze the limiting case of strong affinity differences (α≫1) in the following. The steady state solution of the low-affinity mRNA (Eq. 5) becomes (12)
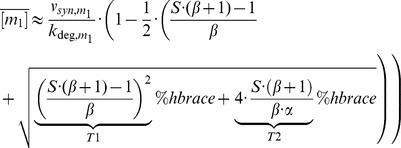
The behavior of the low-affinity species m_1_ is governed by the terms T1 and T2. For low stimuli, the term T1 dominates, and m_1_ will not be significantly affected by sRNA-mediated regulation, .i.e., (13)
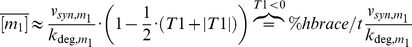
In molecular terms, this regime can be considered to represent sequestration of the sRNA by the high abundance/high-affinity species m_2_. As the stimulus increases, sequestration is less efficient and m_1_ is subject to inhibition as well (term T2 dominates). Thus, sequestration of the sRNA by m_2_ shifts the onset of m_1_ downregulation to higher stimulus levels. Since complete inhibition of both mRNAs is fixed to S = 1, a small fold-change in the input S may be sufficient to switch m_1_ from low to high levels, indicating a strong increase in ultrasensitivity.

Under the assumption that α≫1, we can estimate the onset of m_1_ downregulation by setting T1 = T2≈0, and solving for S. One obtains (14)
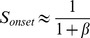
Thus, the larger the expression level of the high affinity mRNA (i.e., the smaller β), the larger the shift in the onset and the more pronounced ultrasensitivity of m_1_. For very low β, *S*_onset_ approaches the stimulus level where the sRNA concentration equals the sum of mRNA concentrations (*S*_onset_≈1), indicating a perfect all-or-none switch. We have thus shown that the switching performance of a threshold-linear sRNA system can be strongly enhanced by the presence of a high affinity competitor, which may either represent another target mRNA or a binding decoy.

Equation 14 represents the extreme case of very large affinity differences (α≫1). In Fig. 2A, we see that enhancement of ultrasensitivity is already observed for moderate values of α and β, although the effects are less pronounced in this intermediate parameter regime. For example, the estimated Hill coefficient as a measure of ultrasensitivity already approaches n_H_≈6 for a ten-fold affinity difference of the two mRNAs (α = 10 and β = 0.1). For comparison, one estimates n_H_≈2 in the single-target case.

Very strong affinity differences between the two mRNAs may imply that the limit of strong binding may no longer hold for the low-affinity mRNA. We therefore performed numerical simulations under the assumption that the low-affinity mRNA no longer shows a threshold-linear response in the single target case (i.e., does not follow a purely stoichiometric mode of inhibition). Importantly, the presence of a high-affinity competitor enhanced ultrasensitivity under these conditions as well (not shown).

To conclude, co-regulation by a shared sRNA gives rise to particularly interesting dynamic behavior if a high-affinity, high-abundance mRNA and a low-affinity/low-abundance mRNA co-exist. In this scenario, co-regulation induces robust synexpression and also enhances ultrasensitivity of the low-affinity mRNA (Fig. 2B).

### Regulation by a shared sRNA synchronizes the temporal induction of mRNA expression

**Figure 3:**
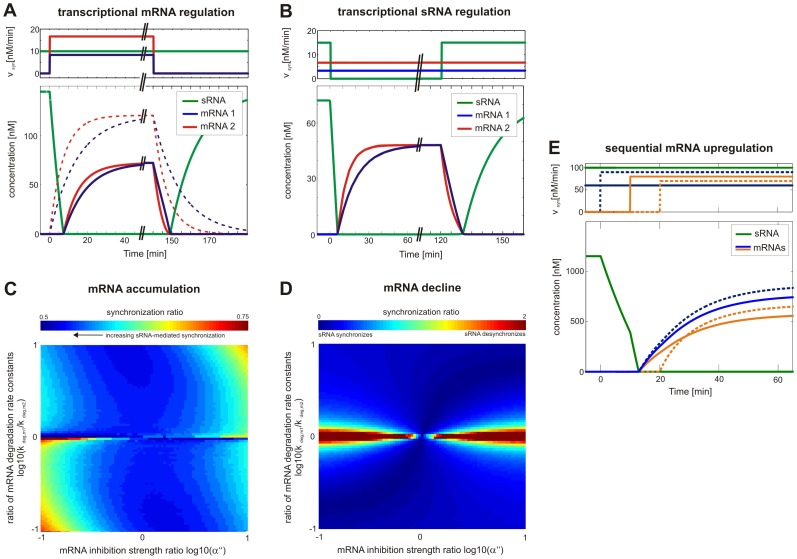
Regulation by a shared sRNA synchronizes the temporal induction of mRNA expression. (A) and (B) Dynamics of mRNA accumulation in response to step-like changes in both mRNA synthesis rates (A) or a step-like change in the sRNA synthesis rate (B). Time-dependent changes in the mRNA or sRNA synthesis rates (v_syn_) are depicted on the top. The solid lines correspond to the sRNA circuit, while the dashed lines depict the behavior of the corresponding sRNA-less system. See Eq. 15 for model equations and Protocol S3 for kinetic parameters. (C) and (D) Synchronous temporal switching occurs mostly independent of kinetic parameters. Response time alignment in the sRNA circuit was compared to the corresponding sRNA-less system using the synchronization factor (Eq. 16). Synchronization factors smaller than unity indicate that sRNA-mediated regulation enhances synchrony in mRNA expression. Synchronization was analyzed as a function mRNA degradation rates and the relative inhibition strength of mRNAs (α″ = k_on,1_/k_on,2_). The heatmaps show simulation results for a regulatory scenario where a coordinate, step-like increase (C) or decrease (panel D) in both mRNA synthesis rates is accompanied by a step-like change in the sRNA synthesis rate in the opposite direction; this counter-regulation assumption was necessary to eliminate delay phases, and to obtain the same steady states in sRNA and sRNA-less systems. Qualitatively similar results are obtained for coordinate regulation of mRNA (but not sRNA) synthesis rates. See Eq. 15 for model equations and Protocol S3 for kinetic parameters. (E) Regulation by a shared sRNAs establishes a delay frame for mRNA induction. Multiple mRNA synthesis rates sequentially increase at different times in a step-like manner (top). Synchronous accumulation of mRNA species occurs upon depletion of the sRNA pool. Late mRNAs whose synthesis starts after the delay period respond immediately due to lack of sRNA buffer (dashed orange line). See Eq. 15 for model equations and Protocol S3 for kinetic parameters.

Our analyses in Fig. 1 indicated that regulator depletion and stoichiometric coupling effects favor synexpression of two mRNAs. Since stoichiometric depletion effects should also occur under pre-steady state conditions, we numerically analyzed whether sRNAs synchronize the temporal dynamics of gene expression as well. For simplicity, the analysis was restricted to a reduced model previously introduced by others [9], [13] (15)
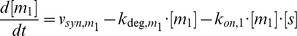


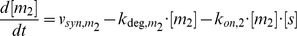


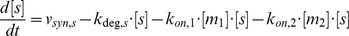
Here, mRNA-sRNA complex formation is assumed to be irreversible owing to high affinity and/or rapid decay of the complex. Numerical analyses of temporal RNA expression were performed to confirm that sRNA-mediated regulation synchronizes the temporal dynamics of gene expression (Fig. 3). We assumed step-like alterations of mRNA or sRNA synthesis rates; these simulations reflect initiation or termination of physiological stress condition where gene expression responses are regulated by altering mRNA or sRNA transcription [2], [25].

Upon step-like upregulation of both mRNA synthesis rates, while sRNA synthesis rate remained constant (at a non-zero value), the multi-target system behaved similarly to the single-target case [7], [8]; sRNA-mediated regulation establishes a delay that equals the waiting time required to deplete the initial sRNA pool (Fig. 3A, left). The duration of the delay phase was exactly the same for both mRNAs despite differences in sRNA affinity and/or expression levels, implying that the onset of mRNA expression is always synchronized. This has important ramifications for synchronizing the expression of mRNAs whose synthesis rates increase in a temporally sequential manner (cf. Fig. 3E and below).

When comparing systems with and without sRNA-mediated regulation, we observe that sRNA regulation can synchronize mRNA accumulation beyond the delay phase as well (Fig. 3A, left). In the sRNA-less system (dashed lines), the accumulation of mRNA is solely determined by mRNA half-lives [42], implying that the short-lived mRNA accumulates faster. In the sRNA system, the difference in mRNA response times can be less than the difference between the half-lives (Fig. 3A, solid lines). This synchronization effect is due to opposite effects on the two mRNAs: Accumulation of the short-lived, high affinity mRNA is slowed down relative to the sRNA-less system (red solid line), since sRNA-mediated degradation efficiently suppresses mRNA accumulation at early (but not late) time points. The long-lived, low affinity mRNA responds more gradually to sRNA-mediated degradation enhancement; the net effect is an increased apparent mRNA turnover rate and thus a shorter response time (blue solid line). We conclude that sRNA-mediated regulation promotes synchronization; it will be shown below that the synchronization effect is particularly pronounced if short-lived mRNA has higher affinity for the sRNA (as assumed in Figs. 3A and B).

Upon coordinated downregulation of both mRNA synthesis rates, we observe a beneficial effect of sRNA-mediated regulation as well (Fig. 3A, right): In the sRNA system, both mRNAs are degraded faster than expected from their half-lives, and this agrees well with the previous observation that sRNA regulation speeds up mRNA downregulation in the single target case [7], [8]. In the multi-target case, this acceleration is beneficial for synchronization and we observe an overlay of three effects: Firstly, enhanced degradation by the sRNA reduces the absolute difference between mRNA time courses, and thereby establishes synchronization. Secondly, once the short-lived, high-affinity sRNA declined to low levels, the sRNA is redistributed to the low-affinity, long-lived mRNA, thereby establishing a coupling effect. Thirdly, mRNA expression is synchronously and completely shut-off once the free sRNA regulator starts to accumulate; this sharpens the decay when compared to exponential kinetics of the sRNA-less system, and further promotes synchronization.

The opposite regulatory case of step-like sRNA synthesis with constant synthesis of both mRNAs is shown in Fig. 3B. We observe similar effects to the case where the mRNA synthesis is regulated (Fig. 3A): Upon a step-like downregulation of the sRNA synthesis, a common delay period is established for both mRNAs (Fig. 3B, left). However, the following accumulation of both mRNAs is solely regulated by their half-life times; thus, sRNA regulation is not beneficial, simply because sRNA is not expressed at all in this scenario. Upon the step-like upregulation of the sRNA synthesis rates both mRNAs are rapidly and synchronously down-regulated (Fig. 3B, right); this behavior is reminiscent of the corresponding scenario in Fig. 3A (right), and arises from degradation enhancement and a sharp shut-off once the sRNA is present in excess.

Summarizing both modes of regulation (Fig. 3A and B), our numerical analyses suggest that three simple rules govern gene expression dynamics of the sRNA circuit: (i) Stoichiometric coupling establishes the same waiting time for all mRNAs upon a shift from no to significant mRNA expression. (ii) The subsequent increase in mRNA species is aligned when the sRNA is further expressed. (iii) The transition from high to no mRNA expression can be efficiently synchronized due to a combination of enhanced degradation and strong stoichiometric inhibition.

To confirm that synchronous rise and shut-down of mRNAs (properties ii and iii) are robust features of the system, we systematically analyzed the parameter space for these scenarios (Fig. 3C and D). We compare sRNA and sRNA-less systems by calculating a synchronization factor (16)
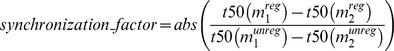
Here, t50 equals the time point where the mRNA concentration has reached half of the difference between initial and final steady states value, and thus measures the response time. The abbreviations m_i_^reg^ and m_i_^unreg^ refer to the sRNA circuit and to the corresponding sRNA-less system, respectively. The synchronization factor thus quantifies synchrony as the absolute difference between the response times of m_1_ and m_2_, and compares sRNA and sRNA-less systems by taking the ratio. Low values of the synchronization factor (<1) imply that sRNA-mediated regulation promotes synchrony of mRNA expression.

For mRNA upregulation kinetics beyond the delay phase, we observe that sRNA-mediated regulation synchronizes expression over a broad range of parameter values (Fig. 3C). As explained in the context of Fig. 3A, the synchronization effect is particularly pronounced if the short-lived mRNA has higher affinity for the sRNA (upper-left and lower-right quadrants in Fig. 3C). Regulation by sRNAs is even more beneficial in coordinating mRNA downregulation, since strong alignment of mRNA time courses is observed for almost all mRNA half-lives and affinity differences (Fig. 3D). Adverse effects of sRNA regulation are restricted to a narrow corridor, where mRNA half-life times are nearly equal and the sRNA-less case already shows pronounced synchrony.

For sRNAs to be effective in synchronizing the temporal dynamics of gene expression in a physiological setting, they should be able to coordinate mRNAs that are induced early and late during cellular stress responses. Numerical simulations were thus performed under the assumption that mRNA synthesis rates sequentially increase in a step-like manner at different times (Fig. 3E). Indeed, sRNA-mediated regulation synchronizes the induction of constitutively transcribed as well as early and late induced mRNAs by establishing a fixed delay time (blue lines and orange solid line in Fig. 3E). However, the synchronization capacity of the system is limited to the time point where the excess sRNA pool is fully degraded; a very late mRNA whose transcription starts afterwards shows no synchronization effect but its accumulation starts without delay (orange dashed line in Fig. 3E).

Taken together, we conclude that shared sRNAs improve temporal synchronization of gene expression by a combination of stoichiometric inhibition and degradation enhancement effects.

## Discussion

Single-input modules (SIMs), where a common regulator controls multiple targets, may exert synexpression or hierarchical prioritization (Fig. 1A). Here, we show that SIM architectures differ strongly in their parameter sensitivity, implying that subtle regulatory differences intrinsically favor synexpression or prioritization. We show that post-transcriptional co-regulation of two mRNAs by a shared sRNA is particularly parameter-insensitive, and thus strongly promotes synexpression without a need for parameter fine-tuning. We numerically confirmed that similar conclusions hold true for the case where more than two mRNAs are strongly regulated by a shared sRNA (not shown). Our results do not contradict previous reports showing that sRNAs can establish hierarchical prioritization [11]: however, we conclude that efficient prioritization would require extremely different affinities for individual mRNAs, at least in the case of high affinity binding.

Small RNAs were previously shown to optimize cellular stress responses by establishing threshold-linear behavior and temporal switching [7], [9]. Cellular stress responses often involve the expression of multiple genes. Our present analyses suggest that sRNAs may be well suited to coordinate the expression of functionally related stress response genes: strong binding effectively suppresses all stress genes under normal conditions, while allowing for highly synchronized all-or-none induction above a critical threshold. Furthermore sRNAs are able to tightly synchronize the temporal induction of multiple genes. Coordinated expression is, however, most pronounced in the regime where the sRNA transcription rate is close to the sum of mRNA transcription rates (near-threshold regime). Thus coordinated expression over a broad expression range by a sRNA is a non-robust property of the system and requires fine-tuning of parameter values. This implies that sRNA-mediated regulation may, for example, not be advantageous to coordinate the levels of constitutively expressed protein complex subunits, without fine-tuning of binding affinities to the sRNA. Previous modeling studies showed that sRNA systems may show large fluctuations in the near-threshold regime [10]. Assuming that noise mostly arises at the level of transcription and that sRNA-mediated regulation is fast, coupling will ensure that functionally related mRNAs fluctuate in a coordinated manner, thus preventing imbalances in cellular regulation.

Two central and experimentally testable predictions can be derived from our model: (i) mRNA pools targeted by the same sRNA may be coupled by sequestration effects, i.e., transcriptional upregulation of one target mRNA should affect the expression of other targets (Eq. 11). (ii) multiple target mRNAs should exhibit synchronous thresholds, independent of precise sRNA binding affinities. The first prediction is well supported by the existing literature, since mRNA targets of bacterial small RNAs and eukaryotic miRNAs appear to communicate with each other by sequestration effects [9], [43]. The second model prediction concerning synchronized gene expression thresholds by post-transcriptional regulation could be verified experimentally by measuring the expression of small RNA targets with different affinities for the common small RNA regulator. Recently work by Hao et al [44] showed that affinities for small RNA regulators and therefore also repression strength can be gradually tuned by sequence alterations in the complementary region of the mRNA and the small RNA. The existence of synchronized temporal thresholds could be confirmed experimentally by measuring temporal kinetics of target expression responses, e.g., after induction of stress. Such temporal responses are already available for multiple targets of the small RNA Spot42 [29]. However, the proposed synchrony effect would require simultaneous analysis of all target mRNAs in the same biological sample.

Our analyses revealed that coupling of mRNA pools by sRNA sequestration effects is the molecular mechanism underlying robust synchronization of mRNA thresholds. Other post-transcriptional regulators like microRNAs or RNA-binding proteins are not degraded alongside with their targets, and thus employ a more catalytic mode of action when compared to bacterial sRNAs. Our unpublished observations suggest that coupling effects are also possible in catalytic system, although the phenomena are less pronounced than in Fig. 2. Accordingly, degradation of multiple proteins by a common degrading activity was recently shown to establish co-regulation of protein levels [45]. Similar to the concepts we present here, co-regulation was especially pronounced when the degrading enzyme was close to its maximum processing capacity (i.e., close to the threshold). We speculate that threshold synchronization by sequestration may synergize with other synchronization mechanisms. For example, it has been show that positive feedback amplifying an upstream regulator synchronizes downstream events in the cell cycle [46]. It is well possible that relatively weak positive feedback may be sufficient to perfectly align pre-synchronized sRNA multi-target systems.

**Figure 4:**
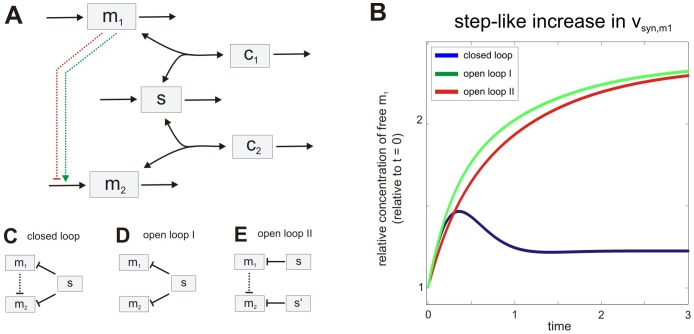
Feedback and adaptation arising from regulation by a shared sRNA. (A) Minimal feedback motif. Feedback regulation arises if the shared sRNA scheme in Fig. 1 is extended such that m_1_ induces or represses transcription of m_2_ (induction: positive feedback; repression: negative feedback); altered levels of m_2_ in turn control the amount of sRNA available for inhibition of m_1_, thus synchronizing the mRNA pools and closing the feedback loop (see text). (B) Adaptation arising from negative feedback regulation. Numerical simulations were performed using the model depicted in (A) assuming m_1_-mediated repression of m_2_ transcription (red arrow). The full system (blue line) shows adaptation to a step-like, 2-fold increase in the m_1_ synthesis rate at t = 0 (v_syn,m1_). In contrast, no adaptation is observed if the feedback loop is broken by removing transcriptional repression of m_2_ (‘open loop I’, panel D) or shared sRNA regulation (‘open loop II’, panel E). Parameters were chosen such that initial m_1_ levels of all topologies are equal (see Protocol S4).

In larger networks, sequestration-based coupling may profoundly affect the system dynamics, as previously discussed for signaling cascades [47], [48], [49], [50], [51], [52]. We expect the same to be true for sRNA networks; for example, a hidden positive feedback and bistability can arise if our minimal reaction network is extended such that m_1_ induces the transcription of m_2_ (Fig. 4A; green arrow) [48]: increasing m_2_ levels reduce the amount of sRNA available for inhibition of m_1_, thus further enhancing m_2_ expression in a positive feedback loop. Similarly, negative feedback can arise if m_1_ represses the transcription of m_2_ (red arrow). Strong negative feedback is known to render biochemical networks insensitive to perturbations [53], [54], [55]. Accordingly, our numerical simulations reveal that the negative feedback model exhibits adaptation which renders the steady state level of m_1_ partially insensitive to the m_1_ transcription rate (Fig. 4B). As expected, no adaptation is observed if sequestration-based feedback regulation by the shared sRNA is removed from the system (Figs. 4B, D, E). We conclude that the synchronization effects discussed in the context of Eq. 10 are sufficient to establish strong feedback regulation in larger post-transcriptional regulatory networks. Sequestration-based negative feedback may contribute to robustness observed in miRNA circuitries [56] which, in one case, was attributed to a single miRNA targeting multiple mRNAs [57]. Detailed simulations of complex post-transcriptional circuitries and quantitative experimental analyses are required to further define the roles of post-transcriptional feedback and feedforward loops in genetic robustness.

## Supporting Information

Protocol S1 **SIM architectures differ in their ability to robustly coordinate gene expression responses (related to** **Fig. 1** **and incl. Fig. S1).**
(DOCX)

Protocol S2 **Analysis of simultaneous switching for independently regulated mRNAs (related to** **Fig. 2** **and incl. Fig. S2).**
(DOCX)

Protocol S3 **Regulation by a shared sRNA synchronizes the temporal induction of mRNA expression (related to** **Fig. 3** **).**
(DOCX)

Protocol S4 **Feedback and adaptation arising from regulation by a shared sRNA (related to** **Fig. 4** **).**
(DOCX)

## References

[1] Aiba H (2007) Mechanism of RNA silencing by Hfq-binding small RNAs. Curr Opin Microbiol 10: 134–139. 17383928 10.1016/j.mib.2007.03.010

[2] Gottesman S (2005) Micros for microbes: non-coding regulatory RNAs in bacteria. Trends Genet 21: 399–404. 15913835 10.1016/j.tig.2005.05.008

[3] Masse E, Escorcia FE, Gottesman S (2003) Coupled degradation of a small regulatory RNA and its mRNA targets in Escherichia coli. Genes Dev 17: 2374–2383. 12975324 10.1101/gad.1127103 PMC218075

[4] Hutvagner G, Zamore PD (2002) A microRNA in a multiple-turnover RNAi enzyme complex. Science 297: 2056–2060. 12154197 10.1126/science.1073827

[5] Babitzke P, Baker CS, Romeo T (2009) Regulation of translation initiation by RNA binding proteins. Annu Rev Microbiol 63: 27–44. 19385727 10.1146/annurev.micro.091208.073514 PMC4682898

[6] Bhattacharyya SN, Habermacher R, Martine U, Closs EI, Filipowicz W (2006) Relief of microRNA-mediated translational repression in human cells subjected to stress. Cell 125: 1111–1124. 16777601 10.1016/j.cell.2006.04.031

[7] Legewie S, Dienst D, Wilde A, Herzel H, Axmann IM (2008) Small RNAs establish delays and temporal thresholds in gene expression. Biophys J 95: 3232–3238. 18599624 10.1529/biophysj.108.133819 PMC2547459

[8] Levine E, Hwa T (2008) Small RNAs establish gene expression thresholds. Curr Opin Microbiol 11: 574–579. 18935980 10.1016/j.mib.2008.09.016 PMC2613760

[9] Levine E, Zhang Z, Kuhlman T, Hwa T (2007) Quantitative characteristics of gene regulation by small RNA. PLoS Biol 5: e229. 17713988 10.1371/journal.pbio.0050229 PMC1994261

[10] Mehta P, Goyal S, Wingreen NS (2008) A quantitative comparison of sRNA-based and protein-based gene regulation. Mol Syst Biol 4: 221. 18854820 10.1038/msb.2008.58 PMC2583084

[11] Mitarai N, Andersson AM, Krishna S, Semsey S, Sneppen K (2007) Efficient degradation and expression prioritization with small RNAs. Phys Biol 4: 164–171. 17928655 10.1088/1478-3975/4/3/003

[12] Mitarai N, Benjamin JA, Krishna S, Semsey S, Csiszovszki Z, et al (2009) Dynamic features of gene expression control by small regulatory RNAs. Proc Natl Acad Sci U S A 106: 10655–10659. 19541626 10.1073/pnas.0901466106 PMC2705582

[13] Shimoni Y, Friedlander G, Hetzroni G, Niv G, Altuvia S, et al (2007) Regulation of gene expression by small non-coding RNAs: a quantitative view. Mol Syst Biol 3: 138. 17893699 10.1038/msb4100181 PMC2013925

[14] Zhdanov VP (2011) Kinetic models of the interference of gene transcription to ncRNA and mRNA. Chaos 21: 023135. 21721777 10.1063/1.3605464

[15] Mukherji S, Ebert MS, Zheng GX, Tsang JS, Sharp PA, et al (2011) MicroRNAs can generate thresholds in target gene expression. Nature Genetics 43: 854–859. 21857679 10.1038/ng.905 PMC3163764

[16] Beisel CL, Storz G (2010) Base pairing small RNAs and their roles in global regulatory networks. FEMS Microbiol Rev 34: 866–882. 20662934 10.1111/j.1574-6976.2010.00241.x PMC2920360

[17] Maki K, Uno K, Morita T, Aiba H (2008) RNA, but not protein partners, is directly responsible for translational silencing by a bacterial Hfq-binding small RNA. Proc Natl Acad Sci U S A 105: 10332–10337. 18650387 10.1073/pnas.0803106105 PMC2492515

[18] Morita T, Mochizuki Y, Aiba H (2006) Translational repression is sufficient for gene silencing by bacterial small noncoding RNAs in the absence of mRNA destruction. Proc Natl Acad Sci U S A 103: 4858–4863. 16549791 10.1073/pnas.0509638103 PMC1458760

[19] Kawamoto H, Koide Y, Morita T, Aiba H (2006) Base-pairing requirement for RNA silencing by a bacterial small RNA and acceleration of duplex formation by Hfq. Mol Microbiol 61: 1013–1022. 16859494 10.1111/j.1365-2958.2006.05288.x

[20] Argaman L, Altuvia S (2000) fhlA repression by OxyS RNA: kissing complex formation at two sites results in a stable antisense-target RNA complex. J Mol Biol 300: 1101–1112. 10903857 10.1006/jmbi.2000.3942

[21] Brantl S (2007) Regulatory mechanisms employed by cis-encoded antisense RNAs. Curr Opin Microbiol 10: 102–109. 17387036 10.1016/j.mib.2007.03.012

[22] Waters LS, Storz G (2009) Regulatory RNAs in bacteria. Cell 136: 615–628. 19239884 10.1016/j.cell.2009.01.043 PMC3132550

[23] Soper TJ, Woodson SA (2008) The rpoS mRNA leader recruits Hfq to facilitate annealing with DsrA sRNA. RNA 14: 1907–1917. 18658123 10.1261/rna.1110608 PMC2525945

[24] Fender A, Elf J, Hampel K, Zimmermann B, Wagner EG (2010) RNAs actively cycle on the Sm-like protein Hfq. Genes Dev 24: 2621–2626. 21123649 10.1101/gad.591310 PMC2994036

[25] Dühring U, Axmann IM, Hess WR, Wilde A (2006) An internal antisense RNA regulates expression of the photosynthesis gene isiA. Proc Natl Acad Sci U S A 103: 7054–7058. 16636284 10.1073/pnas.0600927103 PMC1459017

[26] Papenfort K, Vogel J (2009) Multiple target regulation by small noncoding RNAs rewires gene expression at the post-transcriptional level. Res Microbiol 160: 278–287. 19366629 10.1016/j.resmic.2009.03.004

[27] Lease RA, Cusick ME, Belfort M (1998) Riboregulation in Escherichia coli: DsrA RNA acts by RNA:RNA interactions at multiple loci. Proc Natl Acad Sci U S A 95: 12456–12461. 9770507 10.1073/pnas.95.21.12456 PMC22852

[28] Masse E, Vanderpool CK, Gottesman S (2005) Effect of RyhB small RNA on global iron use in Escherichia coli. J Bacteriol 187: 6962–6971. 16199566 10.1128/JB.187.20.6962-6971.2005 PMC1251601

[29] Beisel CL, Storz G (2011) The base-pairing RNA spot 42 participates in a multioutput feedforward loop to help enact catabolite repression in Escherichia coli. Mol Cell 41: 286–297. 21292161 10.1016/j.molcel.2010.12.027 PMC3072601

[30] Papenfort K, Bouvier M, Mika F, Sharma CM, Vogel J (2010) Evidence for an autonomous 5′ target recognition domain in an Hfq-associated small RNA. Proc Natl Acad Sci U S A 107: 20435–20440. 21059903 10.1073/pnas.1009784107 PMC2996696

[31] Sharma CM, Darfeuille F, Plantinga TH, Vogel J (2007) A small RNA regulates multiple ABC transporter mRNAs by targeting C/A-rich elements inside and upstream of ribosome-binding sites. Genes Dev 21: 2804–2817. 17974919 10.1101/gad.447207 PMC2045133

[32] Gogol EB, Rhodius VA, Papenfort K, Vogel J, Gross CA (2011) Small RNAs endow a transcriptional activator with essential repressor functions for single-tier control of a global stress regulon. Proc Natl Acad Sci U S A 108: 12875–12880. 21768388 10.1073/pnas.1109379108 PMC3150882

[33] Zhdanov VP (2011) Kinetic models of gene expression including non-coding RNAs. Physics Reports-Review Section of Physics Letters 500: 1–42.

[34] Zhdanov VP (2009) Conditions of appreciable influence of microRNA on a large number of target mRNAs. Molecular bioSystems 5: 638–643. 19462021 10.1039/b808095j

[35] Niehrs C, Pollet N (1999) Synexpression groups in eukaryotes. Nature 402: 483–487. 10591207 10.1038/990025

[36] Gurdon JB, Dyson S, St Johnston D (1998) Cells' perception of position in a concentration gradient. Cell 95: 159–162. 9790523 10.1016/s0092-8674(00)81747-x

[37] Hogan DJ, Riordan DP, Gerber AP, Herschlag D, Brown PO (2008) Diverse RNA-binding proteins interact with functionally related sets of RNAs, suggesting an extensive regulatory system. PLoS Biol 6: e255. 18959479 10.1371/journal.pbio.0060255 PMC2573929

[38] Kollmann M, Lovdok L, Bartholome K, Timmer J, Sourjik V (2005) Design principles of a bacterial signalling network. Nature 438: 504–507. 16306993 10.1038/nature04228

[39] Lovdok L, Bentele K, Vladimirov N, Muller A, Pop FS, et al (2009) Role of translational coupling in robustness of bacterial chemotaxis pathway. PLoS Biol 7: e1000171. 19688030 10.1371/journal.pbio.1000171 PMC2716512

[40] Zaslaver A, Mayo AE, Rosenberg R, Bashkin P, Sberro H, et al (2004) Just-in-time transcription program in metabolic pathways. Nat Genet 36: 486–491. 15107854 10.1038/ng1348

[41] Legewie S, Herzel H, Westerhoff HV, Bluthgen N (2008) Recurrent design patterns in the feedback regulation of the mammalian signalling network. Mol Syst Biol 4: 190. 18463614 10.1038/msb.2008.29 PMC2424294

[42] Alon U (2007) Network motifs: theory and experimental approaches. Nature reviews Genetics 8: 450–461. 10.1038/nrg2102 17510665

[43] Salmena L, Poliseno L, Tay Y, Kats L, Pandolfi PP (2011) A ceRNA hypothesis: the Rosetta Stone of a hidden RNA language? Cell 146: 353–358. 21802130 10.1016/j.cell.2011.07.014 PMC3235919

[44] Hao Y, Zhang ZJ, Erickson DW, Huang M, Huang Y, et al (2011) Quantifying the sequence-function relation in gene silencing by bacterial small RNAs. Proceedings of the National Academy of Sciences of the United States of America 108: 12473–12478. 21742981 10.1073/pnas.1100432108 PMC3145688

[45] Mather WH, Cookson NA, Hasty J, Tsimring LS, Williams RJ (2010) Correlation resonance generated by coupled enzymatic processing. Biophysical journal 99: 3172–3181. 21081064 10.1016/j.bpj.2010.09.057 PMC2980735

[46] Santos SD, Ferrell JE (2008) Systems biology: On the cell cycle and its switches. Nature 454: 288–289. 18633407 10.1038/454288a PMC2727670

[47] Bluthgen N, Bruggeman FJ, Legewie S, Herzel H, Westerhoff HV, et al (2006) Effects of sequestration on signal transduction cascades. The FEBS journal 273: 895–906. 16478465 10.1111/j.1742-4658.2006.05105.x

[48] Legewie S, Bluthgen N, Herzel H (2006) Mathematical modeling identifies inhibitors of apoptosis as mediators of positive feedback and bistability. PLoS computational biology 2: e120. 16978046 10.1371/journal.pcbi.0020120 PMC1570177

[49] Legewie S, Schoeberl B, Bluthgen N, Herzel H (2007) Competing docking interactions can bring about bistability in the MAPK cascade. Biophysical journal 93: 2279–2288. 17526574 10.1529/biophysj.107.109132 PMC1965452

[50] Ventura AC, Jiang P, Van Wassenhove L, Del Vecchio D, Merajver SD, et al (2010) Signaling properties of a covalent modification cycle are altered by a downstream target. Proceedings of the National Academy of Sciences of the United States of America 107: 10032–10037. 20479260 10.1073/pnas.0913815107 PMC2890436

[51] Salazar C, Hofer T (2009) Multisite protein phosphorylation–from molecular mechanisms to kinetic models. The FEBS journal 276: 3177–3198. 19438722 10.1111/j.1742-4658.2009.07027.x

[52] Markevich NI, Hoek JB, Kholodenko BN (2004) Signaling switches and bistability arising from multisite phosphorylation in protein kinase cascades. The Journal of cell biology 164: 353–359. 14744999 10.1083/jcb.200308060 PMC2172246

[53] Becskei A, Serrano L (2000) Engineering stability in gene networks by autoregulation. Nature 405: 590–593. 10850721 10.1038/35014651

[54] Fritsche-Guenther R, Witzel F, Sieber A, Herr R, Schmidt N, et al (2011) Strong negative feedback from Erk to Raf confers robustness to MAPK signalling. Molecular systems biology 7: 489. 21613978 10.1038/msb.2011.27 PMC3130559

[55] Paulsen M, Legewie S, Eils R, Karaulanov E, Niehrs C (2011) Negative feedback in the bone morphogenetic protein 4 (BMP4) synexpression group governs its dynamic signaling range and canalizes development. Proceedings of the National Academy of Sciences of the United States of America 108: 10202–10207. 21633009 10.1073/pnas.1100179108 PMC3121836

[56] Li X, Cassidy JJ, Reinke CA, Fischboeck S, Carthew RW (2009) A microRNA imparts robustness against environmental fluctuation during development. Cell 137: 273–282. 19379693 10.1016/j.cell.2009.01.058 PMC2674871

[57] Staton AA, Knaut H, Giraldez AJ (2011) miRNA regulation of Sdf1 chemokine signaling provides genetic robustness to germ cell migration. Nature genetics 43: 204–211. 21258340 10.1038/ng.758 PMC3071589

