# Correction: Multi-Target Regulation by Small RNAs Synchronizes Gene Expression Thresholds and May Enhance Ultrasensitive Behavior

**DOI:** 10.1371/annotation/1bf75d98-6104-4ddf-b897-bfc362f95a5f

**Published:** 2013-12-13

**Authors:** Jörn Matthias Schmiedel, Ilka Maria Axmann, Stefan Legewie

Errors occurred during the preparation of this article for publication. In Equations 12 and 13, the terms '%hbrace' and '%hbrace/t' were added. The correction versions of these Equations can be viewed here: http://www.plosone.org/corrections/pone.0042296.012.cn.tif, http://www.plosone.org/corrections/pone.0042296.013.cn.tif

