# Supplementary material for: Multi-Target Regulation by Small RNAs Synchronizes Gene Expression Thresholds and May Enhance Ultrasensitive Behavior
Source: PLoS One. 2012 Aug 21;7(8):e42296. doi: 10.1371/journal.pone.0042296 (PMC3424230; doi:10.1371/journal.pone.0042296)
Supplement: Protocol S1 — SIM architectures differ in their ability to robustly coordinate gene expression responses (related to Fig. 1 and incl. Fig. S1). (DOCX) [file pone.0042296.s001.docx]

**Supporting Information for “A small RNA targeting multiple mRNA species synchronizes their gene expression thresholds”**

**Protocol S1. SIM architectures differ in their ability to robustly coordinate gene expression responses (related to Figs. 1 and incl. Fig. S1)**

The steady state solution of the minimal cooperative repression model ([m_i_] = V_max,i_ / k_deg,i_ / (K_D,i_^n^ + R_T_^n^)) is proportional to the term describing regulator binding to the promoter (V_max,i_ / (K_D,i_^n^ + R_T_^n^)). We sought to analyze the impact of regulator sequestration on steady state gene expression, and therefore studied regulator binding to the promoter using a more complex model (see Fig. S1 and below). We conclude that regulator sequestration effects render gene expression thresholds less parameter sensitive, and thereby tend to synchronize gene expression responses (cf. Fig. 1B, 1C and S1B).

The simulations in Figure 1C were carried out based on the model depicted in Fig. S1A and using the following set of ordinary differential equations.

Regulator species:

$$\begin{matrix} \frac{ds}{dt} = -k_{on1}*m_{1}*s+k_{off1}*c_{\text{1a}}-k_{on1a}*c_{\text{1a}}*s+k_{off1a}*c_{\text{1b}}-k_{on1b}*c_{\text{1b}}*s+k_{off1b}*c_{\text{1c}} \\ -k_{on2}*m_{2}*s+k_{off2}*c_{\text{2a}}-k_{on2a}*c_{\text{2a}}*s+k_{off2a}*c_{\text{2b}}-k_{on2b}*c_{\text{2b}}*s+k_{off2b}*c_{\text{2c}} \end{matrix}$$

promoter 1 and its complexes:

$$\frac{dm_{1}}{dt}=-k_{on1}*m_{1}*s+k_{off1}*c_{\text{1a}}$$

$$\frac{dc_{1a}}{dt}=k_{on1}*m_{1}*s-k_{off1}*c_{\text{1a}}-k_{on1a}*c_{\text{1a}}*s+k_{off1a}*c_{\text{1b}}$$

$$\frac{dc_{1b}}{dt}=k_{on1a}*c_{\text{1a}}*s-k_{off1a}*c_{\text{1b}}-k_{on1b}*c_{\text{1b}}*s+k_{off1b}*c_{\text{1c}}$$

$$\frac{dc_{1c}}{dt}=k_{on1b}*c_{\text{1b}}*s-k_{off1b}*c_{\text{1c}}$$

promoter 2 and its complexes:

$$\frac{dm_{2}}{dt}=-k_{on2}*m_{2}*s+k_{off2}*c_{\text{2a}}$$

$$\frac{dc_{2a}}{dt}=k_{on2}*m_{2}*s-k_{off2}*c_{\text{2a}}-k_{on2a}*c_{\text{2a}}*s+k_{off2a}*c_{\text{2b}}$$

$$\frac{dc_{2b}}{dt}=k_{on2a}*c_{\text{2a}}*s-k_{off2a}*c_{\text{2b}}-k_{on2b}*c_{\text{2b}}*s+k_{off2b}*c_{\text{2c}}$$

$$\frac{dc_{2c}}{dt}=k_{on2b}*c_{\text{2b}}*s-k_{off2b}*c_{\text{2c}}$$

In the extended cooperativity model (Fig. S1A), the regulator (termed s) binds in multiple steps to the promoters m_1_ and m_2_. Complete promoter inhibition requires binding of three regulator molecules, implying that only the complexes c_1b_ and c_2b_ are inactive, while all other species are actively transcribed (i.e., the active promoter fraction shown in Fig. 1C equals ([m_i_]+[c_i_]+[c_ia_])/ ([m_i_]+[c_i_]+[c_ia_]+[c_ib_]).

This model shows two different switch mechanisms depending on the kinetic parameters: (i) for high-affinity binding of promoter and regulator, stoichiometric switching is observed, i.e., promoters are completely inactivated as soon as the regulator is present in excess over promoter sites (Fig. 1C). (ii) If binding is too weak the promoters are not significantly inhibited even if the regulator is present in excess, i.e., stoichiometric switching fails. However, all-or-none behavior still emerges for even higher regulator concentrations: this is due to the fact that multistep regulation establishes an approximately cubic relationship between amount of regulator and degree of promoter inhibition (‘cooperative switch’; cf. Fig. 1B and not shown). The limiting case of scenario ii is approximated by the minimal cooperative model given in Eq. 1 (main text). While regulator depletion and sequestration effects do not play a role in scenario ii, promoter pools are coupled by regulator sequestration in scenario i.

To introduce differences in the affinity for the regulator between both promoters, we considered the overall dissociation constants to be a function of the inhibition strength ratio $\alpha'$ which takes into account all three binding events.

$$K_{d1}\left(\alpha'\right)=\frac{k_{off1}}{k_{on1}*\sqrt{\alpha'}}*\frac{k_{off1a}}{k_{on1a}*\sqrt{\alpha'}}*\frac{k_{off1b}}{k_{on1b}*\sqrt{\alpha'}}=\frac{K_{d1}\left(\alpha'=1\right)}{\alpha'^{3/2}}$$

$$K_{d2}\left(\alpha'\right)=\frac{k_{off2}*\sqrt{\alpha'}}{k_{on2}}*\frac{k_{off2a}*\sqrt{\alpha'}}{k_{on2a}}*\frac{k_{off2b}*\sqrt{\alpha'}}{k_{on2b}}=K_{d2}\left(\alpha'=1\right)*\alpha'^{3/2}$$

$$\alpha'^{3}=\frac{K_{d2}\left(\alpha'\right)}{K_{d1}\left(\alpha'\right)}$$

We choose the values for the on- and off-rates in Figure 1C as

$$k_{off1}=k_{off1a}=k_{off1b}=k_{off2}=k_{off2a}=k_{off2b}=\frac{1}{min}$$

$$k_{on1}=k_{on2}=0.1\frac{1}{min\cdot nM}$$

$$k_{on1a}=k_{on2a}=1\frac{1}{min\cdot nM}$$

$$k_{on1b}=k_{on2b}=10\frac{1}{min\cdot nM}$$

Our assumption k_oni_ << k_onia_ << k_onib_ ensures that the regulator controls promoter activity in a cubic manner and that the system approaches the minimal cooperative model (Eq. 1; main text) in the absence of regulator depletion.

To establish a stoichiometric switch in Fig. 1C, we chose the parameters such that the target concentrations are large relative to the dissociation constants ($m_{\text{1,}tot}=m_{\text{2,}tot}=100nM$, $K_{d1}\left(\alpha'=1\right)=K_{d2}\left(\alpha'=1\right)=1nM$). Then, all regulator molecules will be bound to the promoters and a threshold is established if promoters and regulator are present in equimolar amounts $s_{tot}\approx3\cdot\left(m_{\text{1,}tot}+m_{\text{2,}tot}\right)$. We also verified that the steady state of the detailed model (Fig. S1A) is proportional to the steady state of the minimal cooperative model (Fig. 1B and Eq. 1; main text) if the target concentrations are small relative to the dissociation constants ($m_{\text{1,}tot}=m_{\text{2,}tot}=0.1nM$, $K_{d1}\left(\alpha'=1\right)=K_{d2}\left(\alpha'=1\right)=1nM$, not shown).

In Fig. S1B, we confirmed our conclusion that regulator depletion effects are required for robust threshold synchronization by systematically analyzing the threshold ratio (Eq. 10; main text) of the detailed model (Fig. S1A). The impact of regulator depletion was analyzed by co-linearly varying both promoter concentrations (m_1,tot_ = m_2,tot_) relative to the dissociation constant of the regulator-promoter complexes (which were assumed to be equal: $K_{d1}\left(\alpha'=1\right)=K_{d2}\left(\alpha'=1\right)=1nM$). The 'degree of regulator depletion' is calculated by taking the ratio of promoter concentration and corresponding affinity ($\frac{m_{\text{1,}tot}}{K_{d1}\left(\alpha'=1\right)}=\frac{m_{\text{2,}tot}}{K_{d2}\left(\alpha'=1\right)}$). For negligible regulator depletion (m_i,tot_ < K_d,mi_) the threshold ratio is sensitive to promoter affinity differences, i.e., changes in α’ (‘cooperative switch regime’, cf. panel B). In contrast, the system is robust for m_i,tot_ > K_d,mi_ (‘stoichiometric switch regime’, cf. panel C). Thus, a sudden transition from asynchronous to synchronous switching occurs as soon as the promoter pools are sufficiently large (y-axis in Fig. S1B); this transition corresponds to the appearance of strong regulator depletion and stoichiometric switching behavior. Taken together, the simulations support that coupling effects due to regulator depletion mask minor parameter differences, and thus synchronize gene expression threshold in a robust manner.


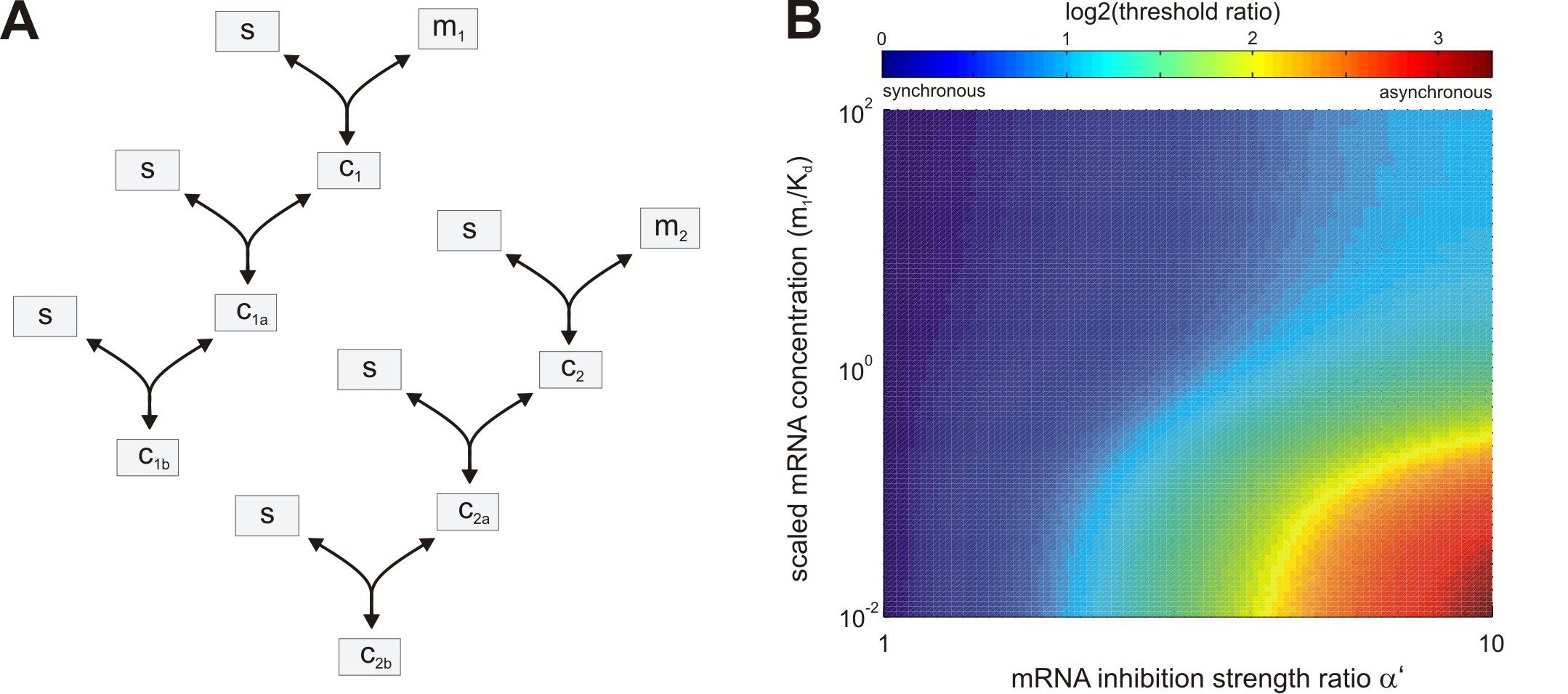


**Figure S1: Cooperative repression model with regulator depletion.** (A) Schematic representation of the model topology (see Supplemental Text for details). (B) Threshold synchronization requires regulator depletion effects. Synchronization was measured by the threshold ratio (Eq. 10, main text). The impact of regulator depletion was analyzed by co-linearly varying both mRNA concentrations (m_1,tot_ = m_2,tot_) relative to the dissociation constant of the regulator-mRNA complexes (see Supplemental text). For m_i,tot_ < K_d,mi_ the threshold ratio is sensitive to mRNA affinity differences, i.e., changes in α’ (‘cooperative switch regime’, cf. Fig 1B). In contrast, the system is robust for m_i,tot_ > K_d,mi_ (‘stoichiometric switch regime’, cf. Fig. 1C).
