# Supplementary material for: Multi-Target Regulation by Small RNAs Synchronizes Gene Expression Thresholds and May Enhance Ultrasensitive Behavior
Source: PLoS One. 2012 Aug 21;7(8):e42296. doi: 10.1371/journal.pone.0042296 (PMC3424230; doi:10.1371/journal.pone.0042296)
Supplement: Protocol S2 — Analysis of simultaneous switching for independently regulated mRNAs (related to Fig. 2 and incl. Fig. S2). (DOCX) [file pone.0042296.s002.docx]

**Supporting Information for “A small RNA targeting multiple mRNA species synchronizes their gene expression thresholds”**

**Protocol S2. Analysis of simultaneous switching for independently regulated mRNAs (related to Fig. 2 and incl. Figure S2)**

In Fig. S2, we investigate synchronous switching capabilities of the system described by Equation 2 in the main text for the case of only one mRNA being transcriptionally regulated, while the synthesis of the other mRNA and the sRNA is constant (i.e. $\beta\neq const.$). We analyze the steady state of the system, which is described by Eq. 4 (see main text).

For our systematic analysis, we choose mRNA 2 to be transcriptionally regulated, while the synthesis rates of mRNA 1 and the sRNA are assumed to be constant. Thus the ratio of mRNA 1 and sRNA synthesis rates is constant as well

$$\kappa=\frac{v_{syn,s}}{v_{syn,m1}}$$

An increase of mRNA 2 synthesis rate can induce a switch in gene expression if and only if the sRNA is more abundant than the unregulated mRNA 1 (by switch we mean here a sudden increase from zero expression to some positive value). In order to focus on such switching, we restrict our analysis to the case where $\kappa>1$.

In the main text, the steady state described in Eq. 5 is determined by the parameters $\alpha$ (mRNA inhibition strength ratio), $\beta$ (ratio of mRNA synthesis rates) and S (stimulus level). For independent regulation of the mRNA synthesis rates,$\beta$and S are both changing; we therefore rescale the input axis to analyze which parameters determine switching of mRNA expression.

For the case of only one mRNA being transcriptionally regulated, the log10-ratio of 10% thresholds of both mRNAs is solely determined by the mRNA affinity ratio $\alpha$ and by the synthesis ratio of sRNA and unregulated mRNA 1$\kappa$(see Supplemental text). Over the whole parameter space, the threshold ratio responds sub-sensitively to parameter changes; the heatmap reveals that robustness of synchronous switching is optimal for$\alpha=1$. For $\alpha\neq1$, synchronous switching is improved if (i) the synthesis of the sRNA exceeds the synthesis of the unregulated mRNA at least by a factor of two ($\kappa$> 2) and (ii) the transcriptionally regulated mRNA has a higher affinity for the sRNA ($\alpha>1$). Our observation that equal or higher affinity of the regulated mRNA ($\alpha>1$) favours synchrony can be explained as follows: If the transcriptionally regulated mRNA has a higher affinity for the sRNA regulator ($\alpha>1$), it efficiently sequesters sRNA away from the unregulated mRNA. Thus, once the regulated mRNA is fully occupied by sRNA, both mRNAs start to be released simultaneously upon a further increase in the mRNA synthesis rate. In the opposite regime (), the transcriptionally regulated mRNA starts to be freed from sRNA, but the high-affinity, unregulated mRNA sequesters the majority of the sRNA pool, and thus remains essentially suppressed (until the transcriptionally regulated mRNA is present in vast excess).


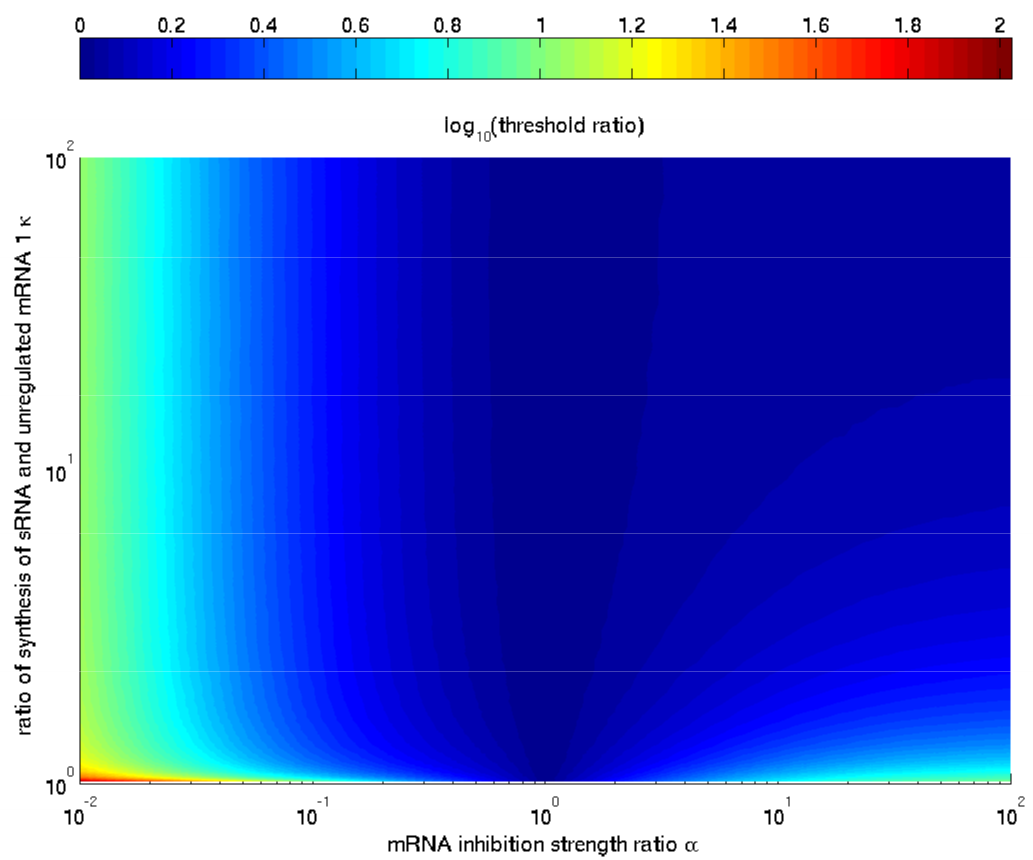
**Figure S2:** Synchronization of gene expression thresholds is maintained for independent transcriptional regulation of co-regulated mRNAs

Since we quantify synchronous switching by a threshold ratio (see main text and below), we can consider$\beta$as the input parameter proportional to v_m2_ (the constant synthesis rate v_m1_ cancels out by taking a ratio of two input levels). Note that S can be rewritten as

$S=\frac{v_{syn,s}}{v_{syn,m1}*\left(1+\beta\right)}=\frac{\kappa}{1+\beta}$,

Thus, the threshold ratio as a measure of synchronous switching

$$threshold\_ ratio=\text{log}_{10}\frac{\beta10\left(m_{1}\right)}{\beta10\left(m_{2}\right)}$$

is solely determined by the parameters$\alpha$and $\kappa$. Similar to the main text, the threshold stimuli$\beta10$are given by the input values (in this case$\beta$), where the full sRNA circuit reaches 10% of the mRNA expression that would be observed in the absence of sRNA; thus$\beta10$is a measure for the input level required to relieve each mRNA from sRNA-mediated suppression.

Figure S2 shows the threshold ratio as a function of the parameters$\alpha$and $\kappa$. Importantly, our analysis above demonstrates that this plot comprehensively characterizes the parameter requirements of the system. Figure S1 thus reveals that synchronous switching over wide parameter ranges is also observed if only one mRNA is transcriptionally regulated.
