# Supplementary material for: Multi-Target Regulation by Small RNAs Synchronizes Gene Expression Thresholds and May Enhance Ultrasensitive Behavior
Source: PLoS One. 2012 Aug 21;7(8):e42296. doi: 10.1371/journal.pone.0042296 (PMC3424230; doi:10.1371/journal.pone.0042296)
Supplement: Protocol S3 — Regulation by a shared sRNA synchronizes the temporal induction of mRNA expression (related to Fig. 3 ). (DOCX) [file pone.0042296.s003.docx]

**Supporting Information for “A small RNA targeting multiple mRNA species synchronizes their gene expression thresholds”**

**Protocol S3. Regulation by a shared sRNA synchronizes the temporal induction of mRNA expression (related to Fig. 3)**

All simulations of the temporal dynamics of the system are carried out using the differential equation system for the reduced model described in Equation 15 (see main text).

**Fig. 3A+B) Numerical analysis of mRNA time courses in the presence or absence of sRNA**

Figures 3A and B show examples of temporal responses to step-like changes in synthesis rates of both mRNAs and the sRNA, respectively. In both simulations the half-life of the sRNA is $t_{1/\text{2,}s}=10min$, the half-life of mRNA 1 (red) is $t_{1/\text{2,m1}}=10min$ and the half-life of mRNA 2 (blue) is$t_{1/\text{2,}m2}=5min$. The coupled degradation rate constant for the interaction between sRNA and mRNA1 is $k_{on1}=0.5\frac{1}{min\cdot nM}$ and the coupled degradation rate constant for the interaction between sRNA and mRNA 2 is $k_{on2}=1\frac{1}{min\cdot nM}$. In both examples the ratio between synthesis rates of mRNA 1 to mRNA 2 is always ½, i.e. the steady states of both mRNAs are always equal. .For the temporal responses to step-like changes in mRNA synthesis rates in Figure 3A, the constant sRNA synthesis rate is $v_{syn,s}=10\frac{nM}{min}$. The synthesis rate of mRNA 1 when up-regulated is $v_{syn,m1}=8.33\frac{nM}{min}$ and the synthesis rate of mRNA 2 when up-regulated is $v_{syn,m2}=16.67\frac{nM}{min}$. For the temporal responses to step-like changes in sRNA synthesis rate in Figure 3B, the constant mRNA synthesis rates are $v_{syn,m1}=3.33\frac{nM}{min}$ and $v_{syn,m2}=6.67\frac{nM}{min}$. The sRNA synthesis rate when up-regulated is $v_{syn,s}=15\frac{nM}{min}$.

**Fig. 3C) Synchronous mRNA up-regulation occurs independently of kinetic parameters**

For our systematic analysis of synchronization of mRNA up-regulation apart from the sRNA-induced delay phase we compared a system where two mRNAs are upregulated once by a step-like increase of mRNA synthesis and once by combined step-like upregulation of mRNA synthesis rates and a simultaneous step-like upregulation of sRNA synthesis (to avoid a delay phase due to before accumulated sRNA pool).

Changes in the ratio of affinity to the sRNA $\alpha$ and changes in the ratio of mRNA degradation rate constants were introduced by varying the coupled degradation rate constant of mRNA 2 (k_on,2_) and the half-life of mRNA 2 (k_deg,m2_) , respectively. The system was induced by increasing both mRNA synthesis rates in a step-like manner at t = 0 min from $v_{syn,m1}\left(t<0min\right)=v_{syn,m2}\left(t<0min\right)=0\frac{nM}{min}$ to $v_{syn,m1}\left(t>0min\right)=v_{syn,m2}\left(t>0min\right)=12.5\frac{nM}{min}$. Further, for the sRNA regulated case, at t = 0 min sRNA synthesis was increased in a step-like manner from zero to $v_{syn,s}(t>0\min)=10\frac{nM}{min}$. The response time of mRNA, defined as the half-maximal time, t50, was quantified by determining the time when mRNA levels had reached 50% of the difference between initial and final steady state levels. Synchronous temporal upregulation by sRNA regulation was quantified using the ratio of 50% threshold time differences between the sRNA regulated and the sRNA unregulated state (Eq. 16; main text).

**Fig. 3D) Synchronous mRNA down-regulation occurs mostly independent of kinetic parameters**

For the systematic analysis of mRNA shutdown we compared a system where two mRNAs are downregulated once by shutdown of mRNA synthesis and once by combined shutdown of mRNA synthesis and simultaneous step-like upregulation of sRNA synthesis. Changes in the ratio of affinity to the sRNA $\alpha$ and changes in the ratio of mRNA degradation rate constants were introduced by varying the coupled degradation rate constant of mRNA 2 (k_on,2_) and the half-life of mRNA 2 (k_deg,m2_) , respectively. The shutdown of the system was induced by decreasing both mRNA synthesis rates in a step-like manner at t = 0 min from $v_{syn,m1}\left(t<0min\right)=v_{syn,m2}\left(t<0min\right)=5\frac{nM}{min}$ (system had reached steady state given synthesis rates for t < 0 min before switching) to $v_{syn,m1}\left(t>0min\right)=v_{syn,m2}\left(t>0min\right)=0\frac{nM}{min}$. Further, for the sRNA regulated case, at t = 0 min sRNA synthesis was increased in a step-like manner from zero to $v_{syn,s}(t>0\min)=20\frac{nM}{min}$. Therefore the system was induced to switch from an expressed to an repressed state. The response time of mRNA, defined as the half-maximal time, t50, was quantified by determining the time when mRNA levels had reached 50% of the difference between initial and final steady state levels. Synchronous temporal upregulation by sRNA regulation was quantified using the ratio of 50% threshold time differences between the sRNA regulated and the sRNA unregulated state (Eq. 16; main text).

**Fig. 3E) Regulation by a shared sRNA regulation establishes a delay frame for mRNA induction**

To show synchronization of temporal thresholds for gene expression despite uncoordinated up-regulation of mRNA synthesis rates, we simulate a system with constant sRNA synthesis, one mRNA also being constantly expressed and three other mRNAs being consecutively up-regulated.

Synthesis rates, up-regulation times, half-lives and coupled degradation rate constants are: constant sRNA synthesis rate: $v_{syn,s}=100\frac{nM}{min}$; constant mRNA 1 synthesis rate: $v_{syn,m1}=60\frac{nM}{min}$; mRNA 2 synthesis rate after up-regulation at t = 0 min: $v_{syn,m2}\left(t>0min\right)=90\frac{nM}{min}$; mRNA 3 synthesis rate after up-regulation at t = 10 min: $v_{syn,m3}\left(t>10min\right)=80\frac{nM}{min}$; mRNA 4 synthesis rate after up-regulation at t = 20 min: $v_{syn,m4}\left(t>20min\right)=70\frac{nM}{min}$; sRNA half-life time: $t_{1/\text{2,}s}=20min$;mRNA half-life times: $t_{1/\text{2,}m1}=t_{1/2,m2}=t_{1/2,m3}=t_{1/2,m4}=10min$; coupled degradation rate constants for all mRNAs:$k_{on1}=k_{on2}=k_{on3}=k_{on4}=10\frac{1}{min\cdot nM}$.
