# Supplementary material for: Multi-Target Regulation by Small RNAs Synchronizes Gene Expression Thresholds and May Enhance Ultrasensitive Behavior
Source: PLoS One. 2012 Aug 21;7(8):e42296. doi: 10.1371/journal.pone.0042296 (PMC3424230; doi:10.1371/journal.pone.0042296)
Supplement: Protocol S4 — Feedback and adaptation arising from regulation by a shared sRNA (related to Fig. 4 ). (DOCX) [file pone.0042296.s004.docx]

**Supporting Information for “A small RNA targeting multiple mRNA species synchronizes their gene expression thresholds”**

**Protocol S4. Feedback and adaptation arising from regulation by a shared sRNA (related to Fig. 4)**

The closed loop system depicted in Fig. 4A (black arrows and red interaction between m1 and m2) was described using the following set of ordinary differential equations

dM1/dt = vsyn_M1-vdeg_M1-von_S_M1+voff_S_M1;

dS/dt = vsyn_S-vdeg_S-von_S_M1+voff_S_M1-von_S_M2+voff_S_M2;

dM2/dt = vsyn_M2-vdeg_M2-von_S_M2+voff_S_M2;

dM1S/dt = -vdeg_M1S+von_S_M1-voff_S_M1;

dM2S/dt = -vdeg_M2S+von_S_M2-voff_S_M2;

where

vsyn_M1=ksyn_M1; % synthesis of M1

vdeg_M1=kdeg_M1*M1; % degradation of M1

vsyn_S=ksyn_S; % synthesis of S

vdeg_S=kdeg_S*S; % degradation of S

vsyn_M2=ksyn_M2/(1+Ki_M1_M2*M1); % synthesis of M2

vdeg_M2=kdeg_M2*M2; % degradation of M2

von_S_M1=kon_S_M1*M1*S; % association of M1 and S

voff_S_M1=koff_S_M1*M1S; % dissociation of M1 and S

vdeg_M1S=kdeg_M1S*M1S; % degradation of M1-S

von_S_M2=kon_S_M2*M2*S; % association of M2 and S

voff_S_M2=koff_S_M2*M2S; % dissociation of M2 and S

vdeg_M2S=kdeg_M2S*M2S; % degradation of M2-S

The following parameter values were used to simulate the initial steady state at t = 0.

ksyn_M1=1; % synthesis of M1

ksyn_S=20; % synthesis of S

ksyn_M2=200; % synthesis of M2

kdeg_S=1; % Degradation of S

kdeg_M2=1; % Degradation of M2

kdeg_M1=1; % Degradation of M1

kdeg_M1S=1; % Degradation of M1S

kdeg_M2S=1; % Degradation of M2S

kon_S_M1=1; % association of S and M1

koff_S_M1=0.1; % dissociation of S and M1

kon_S_M2=100; % association of S and M2

koff_S_M2=0.1; % dissociation of S and M2

Ki_M1_M2=30; % inhibition strength of m2 transcription by m1

The system was perturbed by increasing the synthesis rate of m1 two-fold at t = 0 (i.e., by setting ksyn_M1 = 2).

The open loop model variant I (Fig. 4D) was implemented by eliminating m1-mediated inhibition of m2 transcription (Ki_M1_M2 = 0), and adjusting ksyn_M2 such that the initial concentrations of all molecular species are the same as in the closed loop model variant.

The open loop variant II (Fig. 4D) required a new set of differential equations.

dM1/dt = vsyn_M1-vdeg_M1-von_S_M1+voff_S_M1;

dS/dt = k1*vsyn_S-vdeg_S-von_S_M1+voff_S_M1;

dM2/dt = vsyn_M2-vdeg_M2-von_S’_M2+voff_S’_M2;

dM1S/dt = -vdeg_M1S+von_S_M1-voff_S_M1;

dM2S’/dt = -vdeg_M2S’+von_S’_M2-voff_S’_M2;

dS’/dt = k2*vsyn_S-vdeg_S’-von_S’_M2+voff_S’_M2;

Specifically, the sRNA pool was split into two species (S and S’), each of which selectively inhibits one of the mRNAs. This implies that sequestration effects established by a shared sRNA are eliminated. The new velocity terms read:

vdeg_S’=kdeg_S*S’; % degradation of S’

von_S’_M2=kon_S_M2*M2*S’; % association of M2 and S’

voff_S’_M2=koff_S_M2*M2S’; % dissociation of M2 and S’

vdeg_M2S’=kdeg_M2S*M2S’; % degradation of M2-S

Taken together, the same kinetic parameters were chosen for action of S and S'. Moreover, the parameters were chosen to be equal to the closed loop system, with few exceptions: The synthesis rates of the sRNA pools were adjusted such that the initial concentrations of free and inhibited M1 (i.e., the molecular species M1 and M1S) are equal to the closed loop model variant (k1 = 1/12). The synthesis rate of S’ (k2*vsyn_S) can be chosen arbitrarily.

Numerical simulations reveal that the closed loop model variant adapts to changes in the m1 synthesis rate, while the open loop systems do not, demonstrating that feedback is responsible for adaptation.
